# Recent advances in genetic tools for engineering probiotic lactic acid bacteria

**DOI:** 10.1042/BSR20211299

**Published:** 2023-01-16

**Authors:** Kanganwiro Mugwanda, Saltiel Hamese, Winschau F. Van Zyl, Earl Prinsloo, Morne Du Plessis, Leon M.T. Dicks, Deepak B. Thimiri Govinda Raj

**Affiliations:** 1 Synthetic Nanobiotechnology and Biomachines, Centre for Synthetic Biology and Precision Medicine, Nextgeneration Health Cluster, CSIR Pretoria, South Africa; 2 Department of Microbiology, Stellenbosch University, Private Bag X1, Matieland 7602, South Africa; 3 Biotechnology Innovation Centre, PO Box 94, Rhodes University, Makhanda 6140, South Africa; 4 National Institute for Communicable Diseases, Private Bag X4, Sandringham, Johannesburg 2131, South Africa

**Keywords:** CRISPR, homologous recombination, lactic acid bacteria, probiotic engineering, synthetic biology

## Abstract

Synthetic biology has grown exponentially in the last few years, with a variety of biological applications. One of the emerging applications of synthetic biology is to exploit the link between microorganisms, biologics, and human health. To exploit this link, it is critical to select effective synthetic biology tools for use in appropriate microorganisms that would address unmet needs in human health through the development of new game-changing applications and by complementing existing technological capabilities. Lactic acid bacteria (LAB) are considered appropriate chassis organisms that can be genetically engineered for therapeutic and industrial applications. Here, we have reviewed comprehensively various synthetic biology techniques for engineering probiotic LAB strains, such as clustered regularly interspaced short palindromic repeats (CRISPR)/Cas9 mediated genome editing, homologous recombination, and recombineering. In addition, we also discussed heterologous protein expression systems used in engineering probiotic LAB. By combining computational biology with genetic engineering, there is a lot of potential to develop next-generation synthetic LAB with capabilities to address bottlenecks in industrial scale-up and complex biologics production. Recently, we started working on Lactochassis project where we aim to develop next generation synthetic LAB for biomedical application.

## Introduction

Understanding of the connection between the human microbiome and health has been on the increase and with it, opportunities for developing novel biotherapeutics [1]. This advancement stems from the continuous development of techniques in engineering microorganisms with desired functionalities [2]. Typically, microorganisms that offer inherent beneficial properties to the host are targeted for genetic engineering. Consequently, probiotic microorganisms with improved beneficial properties have been engineered more frequently in recent years.

Probiotics are live microorganisms that confer health benefits on a host when administered in adequate amounts [3]. Most conventional probiotics are gram-positive lactic acid bacteria (LAB) from the genera *Lactobacillus*, *Lactococcus*, and *Bifidobacterium* [4]. These probiotics confer health benefits through diverse mechanisms, including modulating the host immune system, competitive exclusion of pathogenic bacteria, and restoring microbial balance [5]. Conventional probiotics have some drawbacks, including: varied probiotic potential in different hosts, harbouring transferable antibiotic resistance genes, producing non-specific antimicrobials for different pathogens, and producing deleterious metabolites [4,6].

Throughout the years, conventional probiotic bacteria have been genetically engineered to perform unnatural behaviours, enabling various applications. By combining cutting-edge genome modification techniques with novel design concepts, therapeutic systems that go beyond what wild-type microorganisms are naturally capable of can now be designed [7]. This has been facilitated by a shift in the ability to incorporate or alter biological activities and functions in existing microorganisms with inherent desired functionalities [2,4]. Bioengineered probiotic LAB represent a part of the next generation in whole cell-mediated biotherapies for the treatment of human diseases [2,8]. Probiotics are no longer just vectors for the delivery of therapeutics but are now engineered to be microbial ‘physicians’.

Engineered biotherapeutics have several advantages over microbiota-directed approaches such as faecal microbiota transplants [1]. The main advantage is that genetic engineering may confer functions not natively expressed by endogenous microbiota [9]. Engineered probiotics interact with the host mucosal immune system extensively and can be utilised to deliver enzymes, vaccines, antimicrobials, and cytokines [5]. This could provide a more efficient drug delivery system than abiotic therapeutics. Additionally, biotic sensors can be engineered into probiotics for use as non-invasive diagnostic tools [2]. The use of probiotics for drug delivery has mostly been limited to proteinaceous compounds readily synthesised or modified by commensal microbiota [10,11]. Therefore, the continued expansion of the biosynthetic capacities of common probiotics is essential to improve versatility of probiotic-based therapies [2]. Moreover, engineered probiotics that can actively respond to stimuli and that can change their behaviour based on individualised conditions are required [12].

Even though there are extensive benefits of engineered probiotics for therapeutic applications, there are some challenges. The main challenge in engineering probiotics is identifying the most suitable chassis [2]. There is a complex trade-off between survival in the host and safety [10]. A number of engineered *Lactobacillus* species are not part of the resident human microbiota; therefore, they are flushed out by better-adapted microorganisms within days, diminishing their therapeutic effects [13]. Tools for engineering probiotics should extend to resident microbiota to advance engineering of host–microbiota interactions. In this review, we discuss the state of the art in the development of probiotic bacterial chassis, specifically looking at the approaches and tools used in probiotic engineering and heterologous expression systems.

## Engineered probiotic strains

Engineered probiotics are microorganisms with optimised metabolic processes, typically achieved using synthetic biology and omics technologies [4]. Probiotic engineering makes use of bacterial strains that are well suited for colonisation in the gastrointestinal tract and able to produce desired therapeutic molecules *in situ* [14]. The main focus of probiotic engineering is the optimisation of metabolic processes and enhancement of the probiotic potential of microorganisms [4,6].

Understanding the underlying mechanisms of action is critical for developing novel probiotic strains. Probiotics interact with the host and its resident microbiome, optimising microbial composition, competitively excluding pathogens, and degrading toxic compounds [15]. Additionally, probiotic strains interact with the host intestinal mucosa, modulating immune cells and/or increasing epithelial barrier integrity [16]. Probiotic engineering can strengthen existing mechanisms of probiotic action or combine mechanistic pathways found in different strains to produce more potent probiotics.

Bioengineering strategies for probiotics include the design of novel therapeutic approaches to treat infectious diseases, down-regulate autoimmune Type 1 diabetes, and treat cancer (Table 1).

**Table 1:** Examples of engineered probiotics and their expression details

Application	Bacterial chassis	Peptide	Wild-type/Gene source	Expression details	Purpose	Reference
*Antibacterial or Antiviral Activity*	*Lactiplantibacillus plantarum (formerly Lactobacillus plantarum)*	Spike protein Receptor-binding domain (RBD)	Severe acute respiratory syndrome coronavirus 2 (SARS-CoV-2)	Inducible pSIP411 vector	SARS-CoV-2 vaccine	[17]
	*L. plantarum*	SARS-CoV-2 spike protein(S)	SARS-CoV-2	Expression plasmid pLP-tS	SARS-CoV-2 vaccine	[18]
	*Lactococcus lactis*	Reuterin	*Limosilactobacillus reuteri (formerly Lactobacillus reuteri)*	RecT expression vectors; pJP005 andpJP042	Antimicrobial activity	[19]
	*Lacticaseibacillus casei (formerly Lactobacillus casei)*	Listeria adhesion protein (LAP)	*Listeria innocua, Listeria monocytogenes*	Expression vector -pLP401T containing the *pAmy* promoter	Mitigation of lethal *L. monocytogenes* infection	[20]
	*L.casei ATCC344*	Internalins A and B (inlAB)	*L. monocytogenes*	Expression vector-pLP401-T	Prevention of *L. monocytogenes* infection	[5]
	*Lacticaseibacillus paracasei (formerly Lactobacillus paracasei)*	Heavy-chain antibodies (VHHs)	llama	*Lactobacillus* expression vector pLP501	Protection against Rotavirus-induced diarrhoea	[21]
	*Lc. lactis*	Alysteserin, CRAMP1 and Laterosporulin	Synthesised by various bacteria	PTKR vector, *P1* promoter, *usp45* gene	Selective inhibition of *Helicobacter pylori*	[22]
	*Lactobacillus* spp	Highly pathogenic avian influenza (HPAI) virus protein hemagglutinin 1 (HA1)	HPAI virus	*E. coli-Lactobacillus* shuttle vector pLEM415, lactate dehydrogenase (LDH) promoter, *ldhL* promoter	Avian influenza virus vaccine	[23]
	*Lactobacillus gasseri* NM713	Streptococcal M6 protein (CRR6)	*Streptococcus pyogenes*	Expression plasmid-pSLP111.1 based on the xylose operon promoter	Oral vaccine against *Streptococcus pyogenes*	[24]
	*Lactobacillus jensenii*	HIV-1 entry inhibitor cyanovirin-N	Cyanobacterium, *Nostoc ellipsosporum*	Expression cassette containing an *L. jensenii* promoter for the ribosomal protein subunit (PrpsU)	Antiviral activity	[25]
	*Lacticaseibacillus rhamnosus (formerly Lactobacillus rhamnosus)*	Anti-lectin griffithsin	Red algae *Griffithsia* sp.	NICE system, under PnisA control	Targets the HIV virus	[26]
	*Lc. lactis*	Glutamate-rich protein (GLURP)-Merozoite surface protein 3 (MSP3) chimeric protein	*Plasmodium falciparum*	P170 expression system	Malarial vaccine	[27]
*Cancer Therapy*	*Lc. lactis*	KiSS1	Human melanoma cell lines	Expression plasmid pNZ401, nisin inducible promoter	Cancer therapy for prevention of proliferation and migration of human colon carcinoma HT-29 cells	[28]
	*Lc. lactis*	Human Papilloma Virus 16 E7 protein Ag (LL-E7) and biologically active murine IL-12	HPV, murine cells	Nisin inducible promoter	Mucosal vaccine for prevention of HPV Type 16-Induced Tumors	[29]
	*Lc. lactis*	Glycosylated tyrosinase related protein-2	Murine cells	Chinese Hamster Ovary-S cell expression system	Cancer vaccine	[30]
	*L. plantarum*	Oncofetal antigen (OFA)	Mammalian cancer cells	pSIP system, inducible PsppA promoter	Cancer vaccine delivery	[31]
	*Lc. lactis*	Cu/Zn superoxide dismutase	Human cells	PTS System, under strong constitutive P32 control	Defence against carcinogenesis and oxidative damages in the human GIT	[32]
*Metabolic Activity*	*L. gasseri*	Glucagon-like peptide (GLP) 1 (1-37)	Pancreatic β-cell	*SlpA* promoter and *usp*45-LEISS secretion tag	Treatment of Type 2 diabetes	[33]
	*Lc. lactis*	Glucagon likepeptide-1	Pancreatic β-cell	Plasmid pUB1000 used as expression vector	Treatment of Type 2 diabetes	[34]
	*Lc. lactis NZ9000*	Osteocalcin	Murine cell	PnisA control	Diabetes and obesity therapy	[35]
	*L. reuteri*	Phenylalanine lyase	*Anabaena variabilis*	*Lactobacillus* high production constitutive promoter and ribosome binding sequence from the erythromycin resistance B gene (*ermB*)	Treatment of phenylketonuria	[36]
	*Lc. lactis*	Lipase	*Staphylococcus hylicus*.	Nisin inducible promoter	Treatment of pancreatic insufficiency	[37]
Biosensing	*L. reuteri*	Autoinducer peptide-I (AIP-I)	*Staphylococcus* sp	AgrA-activating promoter P3 for controlled expression of the glucuronidase-expressing *gusA* reporter gene	Biosensing AIP-I, a quorum sensing molecule produced by *Staphylococcus* sp. during pathogenesis.	[12]
	*Lc. lactis*	Enterococcal sex pheromone cCF10	*Enterococcus faecalis*	Expression vectors derived from pCF10	Biosensing	[38]
Immune Modulation	*Lc. lactis*	Cathelicidin	Mouse cells	Nisin induced promoter system	Reduction of inflammation in mice with colitis	[28,39]
	*Lc. lactis*	Gliadin	Human cells	*Lc. lactis*-specific pT1NX vector (pT1eDQ8d) used for expression, *Lactococcal* P1 promoter	Immune stimulation	[40]
	*Lc. lactis*	Myelin peptide fragments	Human cells	pIL253 derivative with the *ptcB* gene promoter, expressing MOG35-55, MBP85-97, PLP139-151 antigens	Immune stimulation	[41]
	*Lc. lactis*	Interleukin-10 production	*Yersinia pseudotuberculosis*	Stress-Induced Controlled Expression (SICE) and *usp45* promoter	Trinitrobenzene sulfonic acid (TNBS) induced colitis	[42]
	*Lc. lactis*	Heat-shock protein 65 (Hsp65)	*Mycobacterium leprae*	Xylose-inducible expression system	Prevention of atherosclerosis	[43]

**Figure 1:**
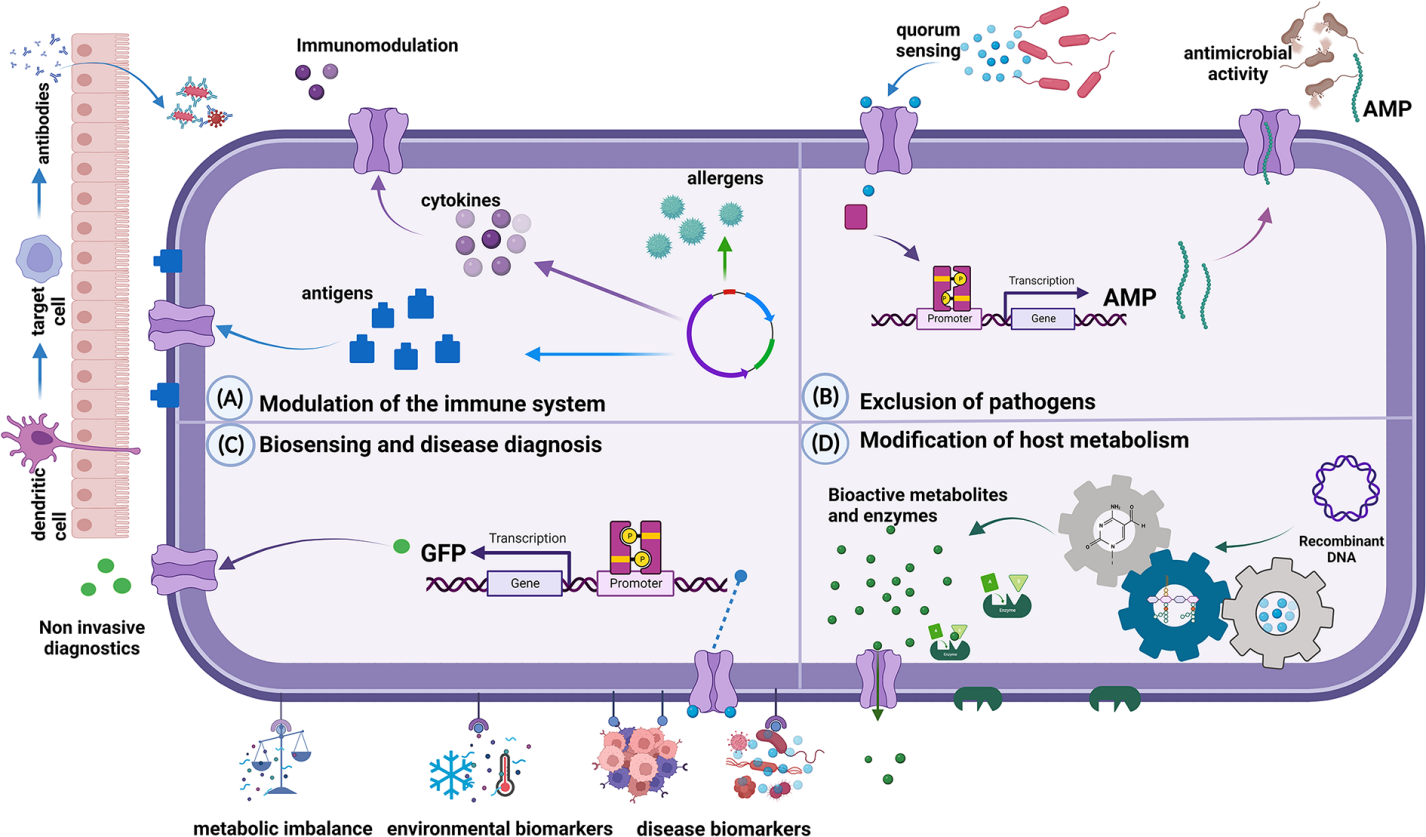
Mechanisms of action of engineered probiotics (**A**) Modulation of the immune system. This may involve stimulation of the production of immunomodulatory cytokines. Engineered probiotics can produce and express antigens that stimulate the production of antibodies. This can be used as a strategy for vaccine delivery. Engineered probiotics may also produce proteins for transcriptional regulation of immune response pathways. (**B**) Exclusion of pathogens due to production of antimicrobial peptides (AMPs) in response to quorum sensing autoinducers produced by pathogenic microorganisms. (**C**) Biosensing and disease diagnosis. Engineered probiotics can detect disease biomarkers, environmental stresses, or metabolic imbalances and elicit a cascade of cellular responses, including regulating gene expression. Detection systems can be coupled to the expression of reporters such as the Green Fluorescent Protein (GFP) to detect and diagnose diseases. (**D**) Modification of host metabolism through production of bioactive metabolites and enzymes. Created with BioRender.com

The novel therapeutic approaches are achieved through mechanisms which include immunomodulation through production of cytokines, antigens, and allergens; exclusion of pathogens through production of antimicrobial peptides (AMPs); biosensing, which can be utilised for disease diagnosis; and modification of host metabolism (Figure 1). These mechanisms have been applied in various therapeutic approaches. For example, a number of *Lacticaseibacillus* species have been engineered to exhibit anti-*Listeria monocytogenes* activity [20,44] or to secrete a recombinant fusion protein of cholera toxin B subunit [45] for the treatment of infectious diseases. Similarly, *Lactococcus lactis* expressing the metastasis-inhibiting peptide KISS1 was developed for cancer therapy [28]. Pusch et al. [46] also engineered recombinant *Lc. lactis* and *Lactiplantibacillus plantarum (*formerly *Lactobacillus plantarum*) to secrete microbiocidal cyanovirin-N to prevent the transmission of human immunodeficiency virus type 1 (HIV-1) and found that it was capable of neutralising the infectivity of HIV-1 *in vitro*. Figure 1 summarises the mechanisms of action of engineered probiotics.

## Synthetic gene circuits and metabolic engineering of probiotics

Synthetic biology approaches can be used to perform targeted genome modifications to rationally design probiotic LAB for robustness, efficiency, and safety [4]. The process for rational engineering of probiotic LAB for therapeutic applications includes (i) the selection of novel health-promoting probiotic strains, (ii) the investigation of the mechanisms underlying their interactions with the host mucosa or resident microbiota at the molecular level, and (iii) engineering them with enhanced and/or designed functional properties based on their inherent probiotic attributes [47]. The goal of the process is to overproduce therapeutic effectors through metabolic engineering, which would have a positive synergetic influence on human health.

In synthetic biology, living cells are designed and constructed from individual components that are purposefully assembled to produce a functional entity [48]. Synthetic biology has enabled the development of tools for the construction of bacterial chassis, including genome editing techniques, expression systems, and gene circuits [49]. This has enabled rational engineering of bacterial chassis, typically achieved by combining genome editing tools with DNA synthesis and assembly technologies [14]. Synthetic gene circuits allow precise regulation of gene expression and the assembly of operons containing synthetic transcriptional and translational control [13]. This permits autonomous decision making, allowing the successful production of biotherapeutics, and ensuring safety.

The term ‘chassis’ is used in synthetic biology to refer to an organism that harbours and maintains the DNA constructs needed for a particular function [50]. A chassis should be able to support the activity of the engineered exogenous genetic components [48,50]. For full functionality, a chassis should have a simplified genome and a metabolic network for synthesis of desired products [2]. To construct a microbial chassis, a combination of computational and molecular tools is used to integrate the components of gene circuits and metabolic pathways. The gene circuits are typically encoded on DNA vectors or plasmids, which are used for transformation of a suitable chassis. An optimised microbial chassis should have robust growth, well-defined metabolic networks, simplified regulation of gene expression, and no evolutionary processes that could impair the functionality of the exogenous gene circuits [48].

A typical synthetic biology workflow applies the Design-Build-Test-Learn cycle to predictably create cells that can produce a wide variety of novel molecules [4]. In the design phase, the chassis is designed by defining the desired outcome and the build components. In the build phase, the generic parts of the chassis are assembled into constructs, and the constructs are introduced into hosts. The test phase involves characterisation of the performance of the chassis [51]. Subsequently, the data from the test phase is used to extract lessons from system performance and conduct an empirical or experimental review of the relationship between sequence and consequences [52,53]. The learning phase will feed into the chassis design and improve the engineering process.

### Construction of probiotic chassis

**Figure 2:**
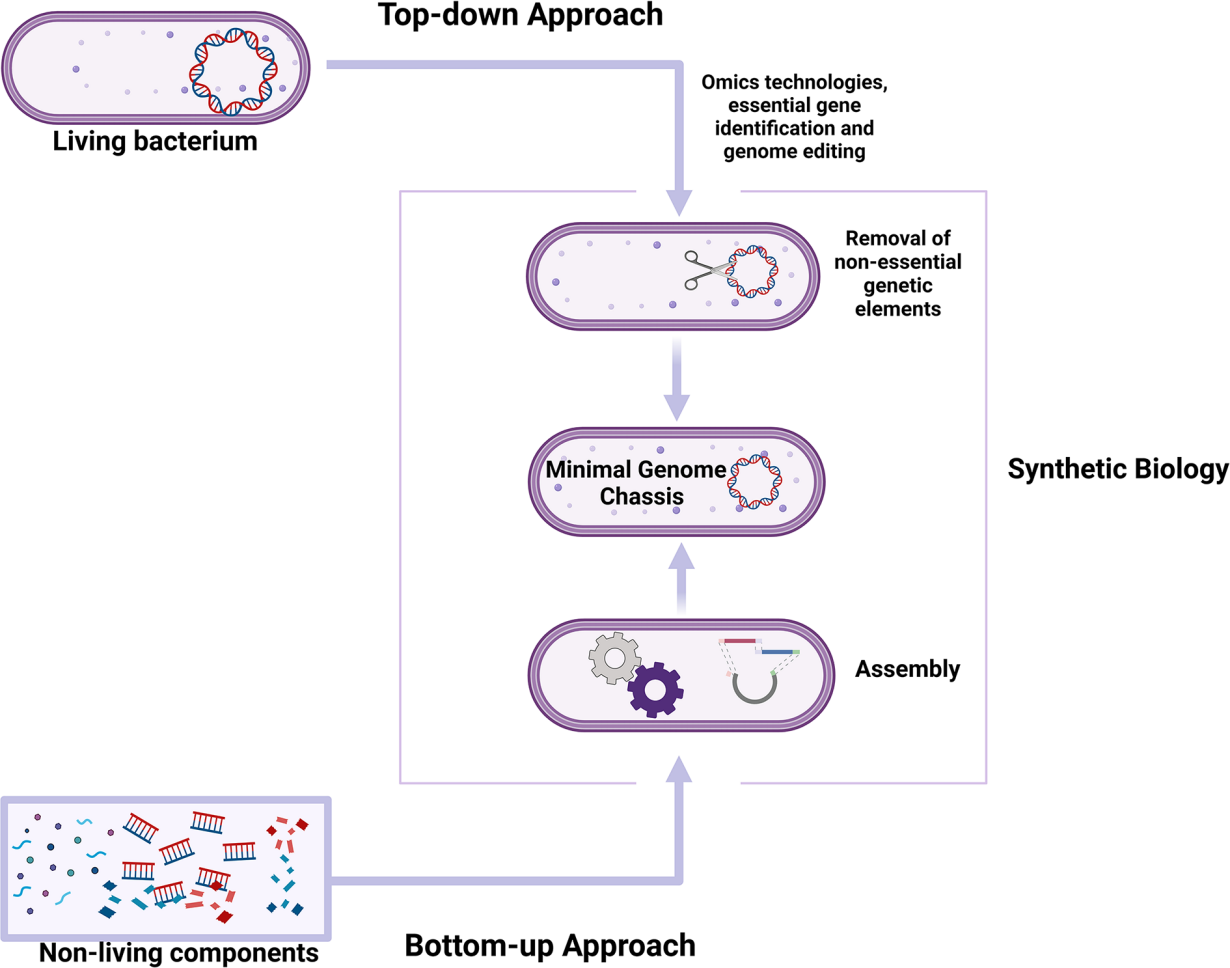
An overview of synthetic biology approaches to probiotic engineering The top-down approach involves identification of essential and non-essential elements and construction of a minimal probiotic chassis by stripping or replacing the genomes of existing probiotics. This approach reduces the complexity of the genome, only retaining the minimum elements essential for viability. In the bottom-up approach, essential, non-living components are synthesised and assembled to construct a probiotic chassis. This approach creates a living minimal probiotic by assembling non-biological and/or non-biological molecules. Created with BioRender.com

A microbial chassis can be constructed using top-down approaches involving genome reduction or bottom-up approaches involving genome synthesis (Figure 2). Either approach can be used to alter metabolic pathways of probiotic microorganisms to add, remove, or modify specific bioactivities using genome editing tools [4]. Top-down approaches to probiotic engineering are thought to be more feasible compared to bottom-up approaches as they can be implemented with incomplete genetic information [54].

#### Top-down approach: genome reduction

Top-down approaches involve the construction of a minimal chassis, often achieved by reducing or simplifying the genome of an existing probiotic (Figure 2). A minimal genome chassis should contain the essential genes and all the necessary components for cell survival [55,56]. The underlying hypothesis in genome minimisation is that removing non-essential genes lowers the cost of maintaining the cell, availing additional resources for exogenous gene circuits and metabolic pathways [48,57]. The phenotypic behaviour of a minimal-genome chassis may be more predictable because of decreased genome complexity. Genome reduction has been shown to boost heterologous protein production and confer significant physiological advantages. Additionally, genome-reduced strains should have improved catalytic efficiency because energy is not wasted in the transcription and translation of non-essential genes [54,57]. The removal of insertion sequences will also likely result in a higher efficiency of electroporation and promote stable integration of exogenous DNA [58]. Overall, genome minimisation will offer several advantages for probiotic engineering.

A major challenge in genome minimisation is cataloguing essential and non-essential genes and functions. Studies on genome reduction and minimilisation show that the majority of the criteria used to determine the essential genes of a bacterial genome are based on functional annotation, comparative genome analysis, and empirically determined gene essentiality categories [59]. Accurate cataloguing of essential genes is critical for probiotic engineering since any viable microorganism should have the minimal set of essential genes [60]. Several tools for cataloguing genes are being developed and optimised for accurate predictions. However, different catalogues of essential genes have been predicted for the same organism using comparable experimental setups [61]. There is a general lack of consensus, making it challenging to determine essential genes in model organisms, and it is even more challenging in non-model organisms. The set of essential genes of an organism is reported to vary significantly depending on the nutrient composition of the medium in which the cells are growing as well as the presence of toxic elements and compounds [57].

Most of the earlier computational gene essentiality prediction tools were based on comparative genome analysis, where essential gene annotations are transferred among related organisms via homology mappings. Currently, machine-learning based prediction is common because essential and non-essential genes for model organisms are listed in publicly accessible databases such as the Database of Essential Genes (DEG) [62], database of essential gene clusters (CEG) [63,64], and Online GEne Essentiality (OGEE) [65]. These databases contain experimental data on gene essentiality and gene properties linked to gene essentiality [66]. Gene essentiality predictions are used to select targets for gene knockouts for genome minimisation projects. Wild-type *Escherichia coli* strains have served as a basis for the construction of several minimal genome bacteria [57,58]. Mizoguchi et al. [67] synthesised reduced genome *E. coli* strains to review the progress of the Minimum Genome Factory project, which was started in Japan in 2001. Their genome-reduced strain outperformed the wild-type *E. coli* strain in terms of growth and threonine production. Since then, there have been several more genome-minimisation projects designed for model microorganisms [49,59,68]. Zhu et al. [69] observed enhanced heterologous protein productivity by *Lc. lactis* NZ9000 when its genome was reduced by 2.83%. In another study, Xin et al. [70] deleted approximately 1.68% of the *Lacticaseibacillus casei* (formerly *Lactobacillus casei*) BLD1 genome. They noted a slightly higher expression of the heterologous green fluorescent protein (GFP) in the genome-minimised strain compared to *L. casei* BL23. Similarly, Qiao et al. [71] deleted the *L. lactis N8* genome by 6.86%. They demonstrated that even though deletion of non-essential regions had no effect on overall nisin yield, it reduced the physiological burden on the strain, thus reducing generation time by 17.18%. However, there have been too few genome-minimisation projects relating to probiotic LAB for definitive conclusions on the overall effect of genome minimisation to be made. This may largely be attributed to challenges in availability of empirical data required for rational minimal genome design for most non-model LAB.

#### Bottom-up approaches: genome synthesis

Bottom-up approaches typically involve the construction of a minimal genome using synthetic oligonucleotides and subsequently inserting it into a cellular envelope to facilitate replication and metabolic activity following transplantation [54] (Figure 2). Construction of a living cell using the bottom-up approach typically requires three basic elements: genetic elements, metabolic systems, and cell membranes [55]. The genetic components are created through *de novo* synthesis and assembly of DNA molecules that correspond to the genomes of existing or designed microorganisms (Figure 2). *De novo* synthesis of entire genomes is now possible, largely owing to Gibson assembly [72], Golden Gate assembly [73], and other DNA assembly technologies. The challenge with the bottom-up approach is that it requires complete information about the core minimal genome of the organism. Additionally, *de novo* synthesis and assembly of long DNA sequences without making errors is difficult, as is transforming the DNA into the chassis [54]. Theoretically, the bottom-up approach can be used to engineer probiotics with novel and predicted properties. However, despite advances made in technologies for *de novo* synthesis of chromosomes, the bottom-up approach is not widely used since optimising is time-consuming and difficult [55].

## Genetic design tools used to engineer probiotics

Genome engineering projects, regardless of scale, require the development of methods capable of introducing precise and planned changes in bacterial genomes [74]. Several tools have been developed for engineering probiotics with varied degrees of effectiveness, precision, and host applicability [75]. In probiotic engineering, genome editing is commonly used to modify bacterial genomes by knocking-out genes, incorporating new genes, or introducing mutations. Genome editing techniques for probiotic LAB are limited in comparison with model bacteria such as *Saccharomyces cerevisiae* and *E. coli*, owing to stringent regulations and poor market acceptance of engineered organisms [1].

### Incorporation of foreign DNA by probiotic microorganisms

The incorporation of foreign DNA, typically in the form of a plasmid, is common in classical genome editing approaches. The foreign DNA is transferred into probiotics via transformation, conjugation, or phage transduction [4,14]. Any genome editing procedure typically begins with transformation, which can be accomplished either naturally or artificially by inducing natural competence, or by disrupting the cell membrane with chemicals, electricity, or other forms of disruption [1]. Natural competence has been observed in several LAB strains [14,76,77]. Competence for natural DNA transformation is induced in response to signalling peptides called competence pheromones [77]. Uptake of foreign DNA by competent cells typically begins with interaction of the cells with double-stranded DNA in their surroundings. This DNA is translocated into the cytoplasm as a single strand. Upon entry, the single strand is bound by proteins such as the recombination protein (RecA) and the DNA processing protein (DprA), and then it is directly integrated into the genome [77]. The mechanisms of activating natural competence are not well understood in most LAB. Typically, researchers introduce foreign DNA into bacteria by inducing competence using various approaches.

One of the most widely used approaches to transferring single-stranded DNA into a recipient bacterium is electroporation. This approach must be preceded by the development of an effective electroporation procedure for the target bacterium. Most electroporation protocols induce competence by culturing cells in hypertonic media with solutions that weaken the cell wall, compromising the natural barrier to exogenous DNA. The competent cells are then subjected to high-voltage electrical pulses that result in the formation of transient membrane pores, allowing negatively charged DNA molecules to enter [77]. The first LAB to be transformed by electroporation in a reproducible and efficient manner was *L. casei* [78]. Since then, transformation protocols have been developed for several other LAB, notably *Lc. lactis*, *L. casei*, *L. plantarum*, and *Levilactobacillus brevis* (formerly *Lactobacillus brevis*) [1,77,79]. According to these studies, most LAB are amenable to genetic modification through electroporation, but strain-to-strain variations in efficiency make it necessary to optimise protocols. In a study by Walker et al. [80], it was determined that electroporation can efficiently introduce plasmid DNA into *Lactobacillus acidophilus* if the integrity of the cell wall is compromised and the osmotically vulnerable cells are protected. The consensus is that transformation efficiency can be improved by optimising parameters such as DNA concentration, voltage, plating regimen, and electroporation buffers.

When electroporation protocols are ineffective, natural methods for introducing DNA into non-model LAB, such as transduction and conjugation, have been proposed as alternatives [77]. Transposons and conjugative plasmids are abundant in LAB genomes. However, their applicability to genome editing has been limited due to poorly understood conjugative mechanisms [1]. Conjugal transfer of plasmids from *Lc. lactis to Lactobacillus delbrueckii subsp. Bulgaricus, Lactobacillus helveticus,* and *Enterococcus faecalis* has been demonstrated [81–83]. Phage transduction is common among LAB, but it is rarely used for targeted DNA exchange. Bacteriophages that infect LAB have been explored extensively because they are a leading cause of fermentation failure in dairy plants [14]. Phage transduction has been used to transfer plasmid or chromosomal genes involved in proteolytic activity, fermentation, bacteriocin production, or antibiotic resistance between LAB. Bacteriophage mediated transduction has resulted in the successful transfer of bacterial DNA between strains with limited genetic accessibility, such as *L. delbruecki*i [84], *Lc. lactis* [85], and other LAB species [14]. Phage transduction is therefore a promising tool that can be used for the engineering of probiotic LAB.

Less commonly used techniques for incorporating foreign DNA into bacteria include sonoporation [86], biolistic bombardment [87], laser irradiation, liposome-mediated fusion [88], and nanofiber piercing [89]. The applicability of these techniques to the incorporation of foreign DNA has been demonstrated; however, their application to new non-model microorganisms is relatively underexplored [90].

### Barriers to uptake and maintenance of foreign DNA by probiotic LAB

To protect against the entry of foreign DNA, bacteria have developed defence mechanisms such as restriction-modification systems, Clustered Regularly Interspaced Short Palindromic Repeat (CRISPR)/CRISPR-associated protein (CRISPR-Cas) systems, or variants of both [1,91]. Approximately 95% of the genome-sequenced bacteria have restriction-modification systems. On average, a bacterial genome encodes two contrasting enzymatic activities for restriction-modification; DNA methyltransferases (MTases) and restriction endonucleases (REases) [92]. The DNA MTases ensure differentiation between self and non-self-DNA by adding methyl groups to a particular DNA sequence within the host genome, whereas the REases recognise and cleave foreign DNA sequences at specific sites [93].

To develop any genome editing technique, it is necessary to bypass host defences against foreign DNA. Three distinct approaches have been proposed to circumvent these defences. These include the use of an intermediate host that is easily transformable and has compatible methylation patterns; the use of a recombinant intermediate host that expresses the methyltransferases that are anticipated to be present in the target microbe; or matching the host DNA methylation patterns through *in vitro* incubation of the DNA with commercial methyltransferases [14]. Incubation with methyltransferases has been used to improve the transformation efficiency of *L. plantarum* to levels comparable to those of *E. coli* [94]. Overall, any genome editing strategy should bypass host defences to ensure efficient incorporation of foreign DNA.

### Gene knock-out and knock-in technologies used for the genetic modification of probiotic LAB

Chromosomal modification strategies have been established for engineering probiotic LAB on the basis of non-replicative plasmids and insertion sequence transposons [77]. Techniques for screening recombinant bacteria and the development of counterselectable markers have advanced rapidly. Genome engineering of probiotics LAB can be accomplished using methods such as homologous recombination, recombineering, and CRISPR-Cas9 mediated genome editing. Additionally, tools such as thermo-sensitive suicide vectors, as well as counter-selectable markers, have traditionally been used to increase the effectiveness of genome editing procedures [1].

#### Homologous recombination

Genome engineering has traditionally relied on homologous recombination (HR). Homologous recombination is typically mediated by sequence-specific programmable nucleases such as the RecBCD nuclease-helicase complex and the RecA single-stranded DNA repair protein. These nucleases are capable of initiating double-strand breaks (DSBs) at targeted gene loci and directing repair-dependent modifications. RecA-dependent recombination occurs rarely and requires long sub-cultivation and laborious screening assays for stable recombinant cells [95]. Double-strand breaks can also be induced at targeted sites using zinc-finger nucleases (ZFNs), transcription activator like effectors nucleases (TALENs), and RNA-guided nucleases, triggering repair [96]. Repairing of the DSB typically facilitates integration of exogenous DNA into the host chromosome. If a homologous donor template DNA guides DSB repairs, this is referred to as homology-directed repair (HDR), and it results in significant changes in the host sequences. If there is no DNA template to direct repair, the non-homologous end-joining (NHEJ) pathway repairs the DSB, typically generating small deletions or insertions [97,98].

Genetic variation in microorganisms may lead to a variety of outcomes when cells develop DSBs. Some LAB are extremely vulnerable to DSBs and may not survive regardless of the repair donor [98]. Earlier, Song et al. [99] had demonstrated that DSBs are lethal to *L. casei* LC2W, *L. brevis* ATCC 367, and *L. plantarum* WCFS1. Genome editing projects for such LAB should be coupled with assistant repair pathways that may potentially enhance repair efficiency.

To use homologous recombination for gene editing, a plasmid with a selectable marker, a counter-selectable marker, and DNA that is homologous to the target gene's upstream and downstream regions is required [90,100]. To stabilise insertional mutations following single-crossover HR, antibiotic selection should be maintained, and multiple mutations cannot be introduced into a strain using the same selection marker. Additionally, insertional inactivation of a particular target may occur within an operon and have polar effects on downstream regions. These constraints can be overcome by creating marker-less gene deletions through a double-crossover HR process that involves plasmid integration and excision [100]. Here, a mutant allele with an internal deletion in the target gene replaces the wild-type allele [101]. The use of counterselectable markers, such as the *upp*, *oroP*, *pheS, and mazF* genes, has led to the development of several modified strategies to edit genomes. Van Zyl et al. [100] used the *mazF* counter-selection marker, a flippase (FLP)/flippase recognition target (FRT) recombination system, and an antisense RNA transcript to develop a novel counter-selection system for chromosomal gene integrations and deletions in LAB. Similarly, Song et al. [102] used *upp* as a counter-selectable marker to enable insertion of target genes in *Lc. lactis* and *L. casei* strains.

#### Suicide plasmids

Genome editing in probiotic LAB has been routinely achieved using suicide plasmid systems. Suicide plasmids can only replicate in the donor bacterium and not in the recipient organism [25,103]. They are known as ‘suicide plasmids’ because they require conditionally defective replication origins. If conditions, such as temperature, change from permissive to non-permissive, suicide plasmids cannot replicate [104]. Suicide plasmids typically contain a homologous sequence with the desired deletion, insertion, or site-directed mutation. Additionally, they contain selectable markers and, on occasion, transposon sequences. Transposon sequences aid in the insertion of the plasmid into the recipient strain’s genome after conjugation [103].

Several studies have reported on the use of suicide plasmids for genome editing in probiotic *Lactobacillus* species. Insertion of genes into various *Lactobacillus* species has been achieved using the pSA3-based suicide vector (pTRK327) containing the IS1223 insert isolated from *Lactobacillus johnsonii* (Table 2) [105]. In a study by Yin et al. [106], a temperature-sensitive suicide plasmid was used to direct chromosomal integration and expression of a porcine rotavirus capsid protein in *L. casei* ATCC 393. They used the *upp* expression cassette for counter-selection (Table 2). The study demonstrated the utility of suicide plasmids for engineering probiotics for vaccine delivery. Similarly, *L. plantarum* gene knockout mutants have been created using suicide plasmids [107]. Ge et al. [108] constructed suicide plasmids to create insertional activation-based gene knockouts in *Lacticaseibacillus paracasei* (formerly *Lactobacillus paracasei*). The recombinant mutant strains were used to study quorum sensing and to predict the impact of targeted gene knockouts on the production of the bacteriocin Paracin 1.7. Suicide plasmids have been successfully used in the integration of heterologous DNA into the genomes of other *Lactobacillus* species [109,110]. Generally, the use of suicide plasmids as a genome editing tool has been limited as a result of very low efficiency and high false-positive rates [103]. This means that several rounds of antibiotic selection are often required to select for mutants. However, suicide plasmids can still be useful when other genome editing tools are not optimised for a specific microorganism.

**Table 2:** Tools for chromosomal editing of probiotic LAB strains

*Genome Editing Tools	Notes	LAB in which application was successful	Reference(s)
*Homologous recombination using the pORI system*	● Homologous recombination via a non-replicating plasmid ● Can be used for gene deletion and insertion of expression cassettes based on conditional replication of vector pORI19	*Lc. lactis, L. acidophilus and L. gasseri*	[111–113]
	Advantages ● Does not dependent on transformation efficiency ● Enables growth of engineered strains at preferred growth temperatures ● Enables efficient recovery of stable integrants ● Applicable to deleting any non-essential gene across a broad range of species ● Seamless genome editing		
	Limitations ● Plasmid DNA and antibiotic markers remain integrated in the chromosome, this complicates applications ● Time consuming and laborious		
pTRK system	● Site-specific chromosomal integrations and deletions	*L. acidophilus, L. gasseri, L. casei, Lc. lactis, L. plantarum*	[114,115]
	Advantages ● Host temperature range that includes thermophilic lactobacilli ● Independent of transformation efficiency ● Can be used for marker-less gene replacement by using *upp* as counter-selectable marker for positive selection of double recombinants		
	Limitations ● The stability of the insertional mutations after single-crossover HR requires maintenance of antibiotic selection ● The same selection marker cannot be used to introduce multiple mutations into a strain ● Insertional inactivation of a specific target within an operon may have polar effects on downstream region		
Cre-*lox* system	Can be used for deletions and insertions	*L. plantarum, L. casei, L. lactis*	[69,70,116]
	Advantages ● Allows the removal of selectable marker(s) upon marker selection of deletion variant		
	Limitations ● The presence of multiple loxP sites recognisable by Cre might lead to genome instability		
ssDNA recombineering	● Homologous recombination of single- stranded linear DNA utilising λ-Red enzymes Gam, Exo, and Bet ● Targeted chromosomal mutation ● When assisted by RecT-mediated recombination, mutagenesis efficiencies of 0.4 to 19% can be achieved	*L. reuteri, Lc. lactis, L. plantarum, L. gasseri*	[19]
	Advantages ● Site specific ● Not hyper mutagenic ● Efficient for subtle genome modifications ● Allows selection of mutants without antibiotic marker selection		
	Limitations ● Recombineering efficiency is dependent on expression of RecT homologs ● Inefficient for large chromosomal modifications (>1 kb) ● Selection of the desired mutations can be laborious and time consuming		
dsDNA recombineering	● Recombinase-mediated deletions and insertions	*L. plantarum, L. casei, L. paracasei*	[117,118]
	Advantages ● Enables manipulation of large genomic regions ● Easy screening of mutants ● High efficiencies for both deletion and insertion		
	Limitations ● Efficiency is dependent on specific interactions between recombinases and host-encoded proteins ● Antibiotic selection is required for higher efficiency of genome editing ● Removal of antibiotic markers employs Cre/*loxP* leaving a lox scar on the genome		
CRISPR-Cas9	Precise genome editing using CRISPR/Cas9	*Lactobacillus crispatus, L. plantarum, L. reuteri*	[101,119]
	Advantages ● Enables programmable, precise genome editing ● High efficiency (up to 100%) for small deletions ● Marker free selection ● Have multiplexing potential, several deletions or genome modifications can be performed concurrently		
	Limitations ● Transformation independent ● Limited to genetic sites with protospacer adjacent motif (PAM) motifs present ● Can introduce lethal double-strand breaks in off-target sites		
ssDNA recombineering + CRISPR-Cas9	ssDNA recombineering combined with CRISPR/Cas9 for targeted chromosomal mutations	*L. reuteri, Lc. lactis, L. gasseri, L. plantarum*	[95,120,121]
	Advantages ● Allows genome editing without relying on restriction enzymes or antibiotic markers ● Introducing a CRISPR-Cas9 plasmid into ssDNA-recombineered bacteria can eliminate many of the unedited cells and improve the efficiency to >75% ● Time efficient (workflow can be completed within 72 h)		
	Limitation ● Inefficient for large chromosomal modifications (>1 kb)		
dsDNA recombineering + CRISPR-Cas9	Used to generate point mutations, deletions, insertions, and gene replacements	*L. plantarum, L. brevis*	[98,120,122]
	Advantages ● Effective, precise genome editing ● Seamless genome editing (sgRNA removes the *loxP* site)		
	Limitations ● Lactobacilli respond to CRISPR-Cas9‐induced DSBs in a differently; therefore, the efficiency is difficult to predict ● A DSB or a nick may be ineffective in triggering HDR, resulting in cell death		
CRISPR-Cas9^D10A^ Nickase-assisted plasmid toolbox (pLCNICK)	Gene deletion and insertion using the Cas9D10A (nickase) ● Efficiency for deletions and insertions between 25 to 62% ● Correlation between deletion size and efficiency	*L. casei, L. acidophilus, L. gasseri, and L. paracasei*.	[99]
	Advantages ● Efficient, rapid, and precise tool for genome editing in *L. casei* ● Can circumvent DSB-induced lethality, probably due to variant repair pathways of nicks ● Marker free		
	Limitations ● Inefficient for large deletions ● High fatality due to Cas9/sgRNA-induced DSBs		
CRISPRi	Incorporation of dCas9 nuclease and sgRNA into the chromosome is required for genome editing	*L. plantarum, Lc. lactis*	[101,123,124]
	Advantages ● Repression of single or multiple target genes simultaneously ● dCas9 has an easily replaceable 20-nucleotide base-pairing region that can be programmed to target any gene of interest ● Enables easy down-regulation of any gene of interest ● Reversible effects ● Marker free ● Easier screening tool ● High potential for multiplexing ● Can be used for essential gene studies		
	Limitations ● Silencing of non-target genes ● High levels of dCas9 expression coupled with off target binding of sgRNA can be toxic to cells ● CRISPRi system is active even without induction, this could affect essential genes and result in slower growth		
Cre-*loxP* system	Site specific deletions, insertions, translocations, and inversions at specific sites	*Lc. lactis, L. plantarum*	[69,70,116]
	Advantages ● Can be used for simplified and programmable construction of large-scale chromosomal deletions (up to 39 kb) ● Very effective and precise due to the high affinity of the Cre recombinase for l*oxP* sites ● Enables marker-less deletion, no need for counter selection ● Can be used for sequentially generating multiple deletions		
	Limitations ● Very laborious and time consuming because it involves extensive selection and screening ● Cre*/loxP* carries the risk of creating unwanted effects at non target sites, and can be mutagenic		
Selection/counter-selection marker system	Use of *upp* or *mazF* for chromosomal gene deletions, integrations, and deletions in LAB	*L. plantarum 423, Enterococcus mundtii ST4SA, L. casei, Lc. lactis, L. acidophilus*	[100,102,114]
	Advantages ● Efficient deletion or integration of genes at specific loci ● The *upp* counterselectable marker is recyclable ● Resulting in transgenic or mutant strains do not contain any selectable markers or residual plasmids ● Enables construction of stable double-crossover mutants		
	Limitations ● The *upp* gene is involved in the nucleotide metabolic pathway of almost every organism ● 5-fluorouracil (5-FU) may be toxic, even in *upp* mutants; this complicates the use of counterselection for heterologous upp expression ● Identifying and optimising suitable counter-selection markers can be challenging and laborious.		

#### Recombineering

**Figure 3:**
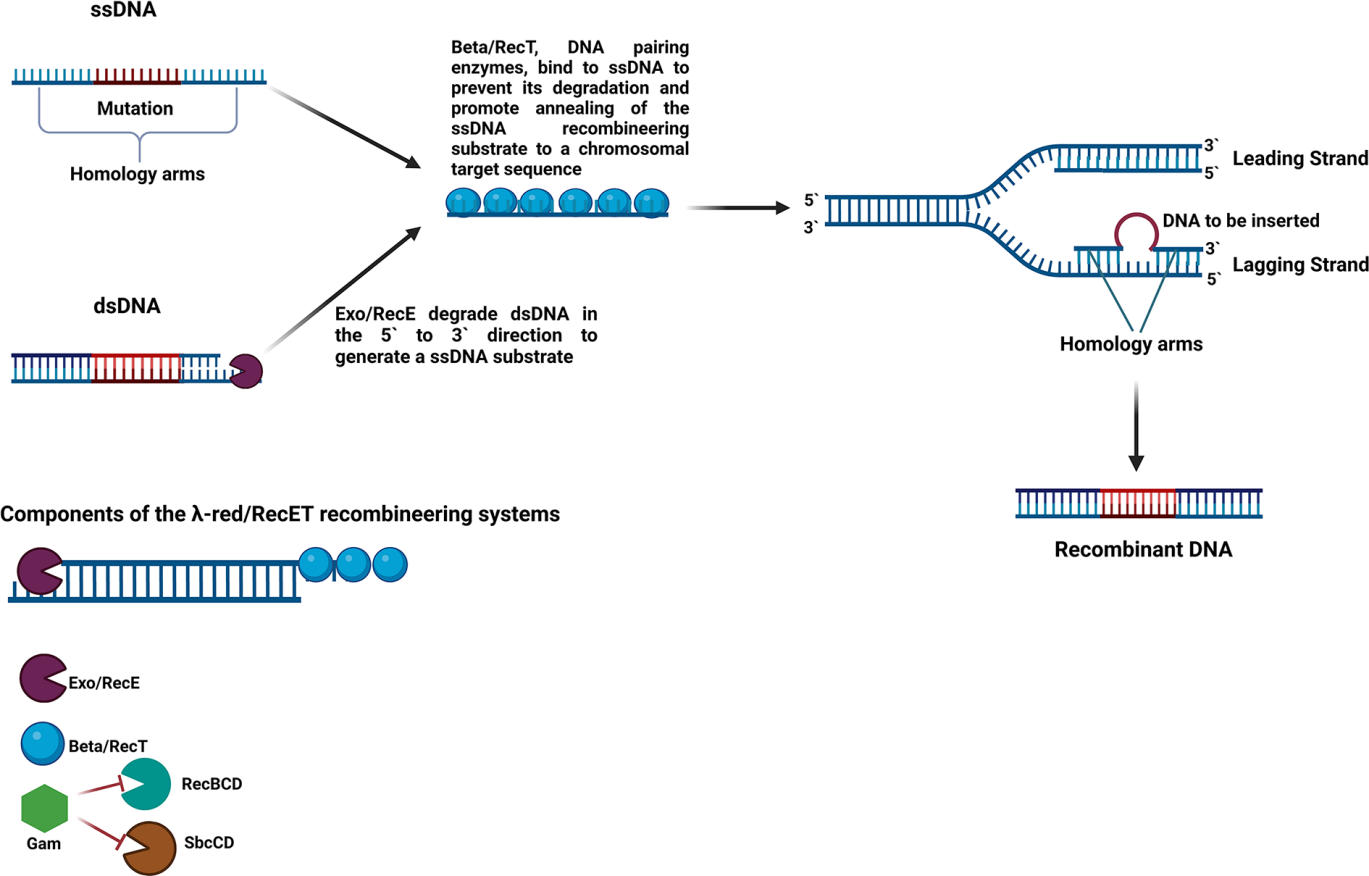
Genome editing using the three components of the λ-red recombineering system with Exo, Beta, and Gam or the RecET recombineering system with recT and recE The editing DNA fragments may be either single-stranded (ssDNA) or double-stranded (dsDNA). When the editing DNA is double-stranded, Exo/recE degrade one strand from the 5′ direction to the 3′ direction, generating a ssDNA substrate. Beta or its Rac analogue, RecT bind the ssDNA and prevent its degradation by endogenous nucleases while being escorted to a replication fork. Additionally, Beta/RecT promotes annealing of the editing ssDNA to a target site on the lagging strand. Annealing of the editing ssDNA to the target is facilitated by the homology arms flanking the desired edit. The editing ssDNA acts as an Okazaki fragment. Gam prevents digestion of DNA by endogenous RecBCD and SbcCD and increases recombineering efficiency. Created with BioRender.com

Recombineering is a phage-based system that achieves gene deletion, insertion, or replacement through *in vivo* homologous recombination (HR) between exogenous DNA and the host genome [105,125]. The term ‘recombineering’ is used to describe genetic engineering mediated by HR [126]. Recombineering is possible using double-stranded (dsDNA) or single-stranded DNA (ssDNA), and is facilitated by Red/RecET systems [77] (Figure 3). The Red system relies on the expression of three proteins derived from the lambda (λ) phage: Beta, Gam, and Exo. RecET proteins are derived from the Rac prophage and are encoded by two genes that are adjacent to each other, *recE* and *recT*. These proteins are collectively referred to as recombinases [117]. RecE and Exo degrade dsDNA in the 5′-3′ direction, generating 3′-ended ssDNA overhangs whereas RecT and Beta anneal to ssDNA and promote annealing of complementary DNA strands, as well as strand exchange and invasion [19,118] (Figure 3 and Table 2).

The Red/RecE system has enabled precise genome editing in model microorganisms such as *E. coli*. However, there has been little success using the Red/RecET system on bacteria other than *E. coli*, possibly due to the need for specific interactions between the recombinases and the proteins that are encoded by the host [120]. More recent applications of recombineering use ssDNA as a substrate rather than dsDNA. Single-stranded DNA recombineering has been developed in *Limosilactobacillus reuteri* (formerly *Lactobacillus reuteri)*, *Lactobacillus gasseri*, *L. casei*, and *L. lactis*, allowing subtle genome modifications such as point mutations [19,127] (Table 2).

Single-stranded DNA recombineering enables the engineering of subtle chromosomal mutations without the use of selectable markers. The use of ssDNA recombineering requires the capacity to transform an oligonucleotide with the desired modifications into a suitable host cell and to induce the production of recombinases such as RecT or Beta. The efficiency of recombineering can be improved using CRISPR-Cas9 assisted genome editing [105]. The use of ssDNA recombineering in conjunction with CRISPR-Cas9 to select for recombinant bacteria means that genetic engineering will become increasingly viable without the use of antibiotics as selection markers. The discovery of new Cas9 proteins with distinct protospacer adjacent motifs (PAMs) will also increase the number of genome-wide target sites for genome engineering [127].

To enable high-throughput genome editing, a recombineering technique known as Multiplex Automated Genome Engineering (MAGE) was established [128]. This method improves upon the traditional λ-Red recombinase-based recombineering. High-throughput recombineering can be achieved using the λ-Red recombinase system and a pool of oligonucleotides to rapidly introduce simultaneous genome modifications [75].

Recombineering can also be achieved using site-specific recombinases such as the Cre-*lox* system (Table 2). This system is highly efficient and functional in many organisms [70,116]. The Cre-*loxP* system is based on the P1 bacteriophage and has two components: the Cre recombinase, which facilitates site-specific recombination; and two *loxP* sites, which facilitate excision of target DNA. *LoxP* sites are 34 bp long sequences that contain 13 bp long palindromic recognition sites separated by an 8 bp spacer region. The Cre recombinase excises any chromosomal region flanked by two *loxP* sites in the same orientation [70]. The Cre-*loxP* system has been successfully used for genome editing in *Lc. lactis*, *L. plantarum* (Table 2) [129,130]

#### CRISPR-Cas9 assisted genome editing

**Figure 4:**
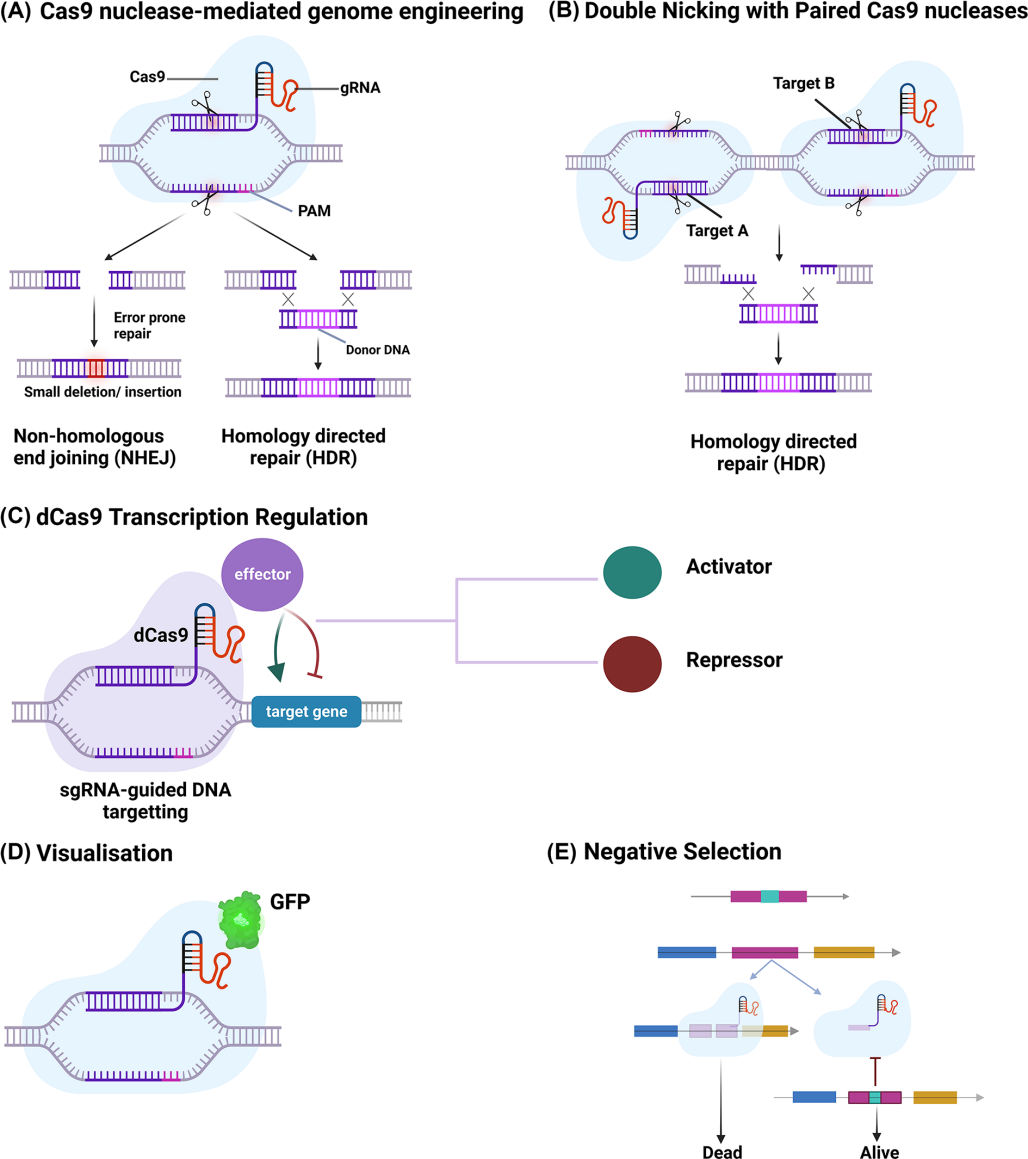
An overview of CRISPR applications in genome engineering (**A**) Cas9 nuclease-mediated genome editing relies on Cas9 cleaving target DNA. The Protospacer Adjacent Motif (PAM) enables Cas9-mediated recognition and cleavage of target DNA. (**B**) Double nicking with paired Cas9 nucleases for genome engineering. (**C**) Catalytically ‘dead’ Cas9-variant (dCas9) transcription regulation. It may be CRISPR activation (CRISPRa) or CRISPR interference (CRISPRi). The transcription regulation relies on the use of dCas9, a mutant form of Cas9 that can bind but not cleave DNA. dCas9 controls gene expression by inhibiting transcription. (**D**) CRISPR-based visualisation. (**E**)CRISPR-based negative selection. Cas9 can be used to cleave unmodified DNA for negative selection. Created with BioRender.com

The CRISPR-Cas9 system system has revolutionised genome editing, providing sequence-specific targeting of DNA [131,132]. Probiotics can be genetically modified to increase their probiotic properties using both endogenous and engineered CRISPR-Cas systems (Figure 4). The Cas9 endonuclease can drive binding and cleavage of DNA through an engineered single guide RNA (sgRNA) sequence. This enables the simultaneous delivery of recombination templates and self-targeting templates on plasmids to modify genotypes and produce mutations, deletions, and insertions. The CRISPR-Cas systems are simpler to repurpose for genome editing since they rely solely on base-pairing of the sgRNA with the target sequence [].

CRISPR-Cas systems are abundant in LAB, particularly in *Bifidobacteria* and *Lactobacillus*, because of the frequent exposure of these bacteria to bacteriophage DNA or foreign plasmids present in natural environments such as fermented foods and the gastrointestinal tract (GIT) [4,133]. Type II CRISPR-Cas systems are unusually common in *Lactobacillus* species and have received the most attention because of the Cas9 signature nuclease's capacity for precise, programmable, and efficient genome editing [133]. Exogenous CRISPR-Cas systems can be introduced using plasmid-based systems when probiotic strains lack CRISPR-Cas systems or they are ineffective [134]. CRISPR-Cas assisted genome editing has been applied to *Lactobacillus reuteri* ATCC PTA 6475 [121], *L. plantarum* [120], and several other LAB (Table 2). Song et al. [99] developed the pLCNICK plasmid based on CRISPR-Cas9^D10A^ for rapid and precise editing of the *L. casei* genome (Table 2). This pLCNICK plasmid has a broad host range and can be adapted for genome editing of other *Lactobacillus* species. The use of functional CRISPR-Cas9 plasmid delivery is crucial, especially in LAB that lack active CRISPR-Cas9 systems, such as *L. acidophilus*. Several Cas9-nucleases and CRISPR-Cas systems are being explored for use in bacterial genome engineering, with the goal of achieving wider applicability, precision, stability, and lower toxicity [1]. An overview of CRISPR applications in genome editing is shown in Figure 4.

Leenay et al. [122] determined that the outcome of genome editing using CRISPR-Cas9 in *L. plantarum* is dependent on the method and strain used. This variation was attributed to features that differentiate comparable strains, including the efficiency of transformation, DNA repair protein expression and activity, or the likelihood of genomic excision events. CRISPR-Cas systems are designed to prevent the transfer of exogenous DNA into host genomes [131]. This can be exploited to precisely prevent the spread of genetic elements, such as antibiotic-resistance markers, toxins, and other detrimental genes between bacterial strains. It could also be designed to control the spread of mobile genetic elements like insertion sequences and transposons within cells, ensuring greater genetic stability [135]. Although the CRISPR-Cas system is a potent tool for genome engineering, it is frequently challenging to optimise in non-model bacteria [90]. CRISPR-mediated genome editing is typically coupled with other techniques to optimise the efficiency of genome editing. In a study by Huang et al. [98] RecE/T-assisted CRISPR-Cas9 editing was found to be highly efficient in *L. plantarum* WCFS1 and it was inferred that this system could be applied to other *Lactobacillus* species (Table 2).

#### Random DNA insertion

Genome engineering in non-model organisms may make use of non-homology-based approaches. A widely used approach is the use of transposons that enable the random integration of DNA segments across the genome without the need for homology [90]. Examples of transposons used for random DNA insertion include *Himar1*, which inserts randomly between any TA dinucleotide, and Tn5, which can insert in the genome mostly at random but has a bias towards certain DNA shapes and GC rich or AT rich regions [136,137]; therefore, Tn5 insertion is not uniformly random. The combination of random transposon mutagenesis and high-throughput sequencing for genome-scale analyses is referred to as transposon sequencing (TnSeq). This approach is typically used to study bacterial gene function and inform targets for genome engineering. Transposons may also be used to introduce new pathways into bacteria. It is possible to insert a landing pad using the Cre-*lox* site-specific recombination system in conjunction with transposon mutagenesis before introducing heterologous pathways at specific sites [90]. Random transposon mutagenesis has been used successfully in the engineering of *L. plantarum* [138] and *L. casei* [139]. However, random insertion of DNA into the genome of an existing probiotic can be problematic. The transposon frequently integrates into the coding regions, which could affect strain fitness. Additionally, identifying the location of the transposition needs considerable effort. Variations in the level of gene expression of the same construct can also be produced by random integration [90]. Overall, tools for genome editing are not mutually exclusive and are usually combined depending on the host species and desired outcome. Table 2 summarises chromosomal editing tools used to produce engineered probiotics.

## Heterologous protein expression in engineered probiotics

Heterologous protein expression typically involves using expression systems from native cDNA clones or using DNA technology products such as synthetic oligonucleotides and designer proteins. The high level of genetic diversity in LAB species makes it difficult to prepare broad application expression vectors for gene cloning and expression. Some replication systems are only functional in particular strains, and known lactobacilli and lactococcal promoters exhibit varying levels of activity in different strains [140]. Several heterologous expression systems have been investigated to produce engineered probiotic strains (Table 1).

Expression systems used in heterologous expression of proteins by engineered probiotics may be constitutive or inducible. Constitutive expression may result in overproduction and accumulation of proteins that have a detrimental effect on cells [141,142]. As a result, novel controlled-expression systems must be engineered [143]. These systems are designed to protect the bacterial chassis against the potentially harmful effects of overproduction of heterologous proteins. This is accomplished by controlling gene expression via external environmental factors including temperature, pH, bile salt concentration, or the presence of antimicrobial peptides [141].

### Commonly used expression systems in LAB

**Figure 5:**
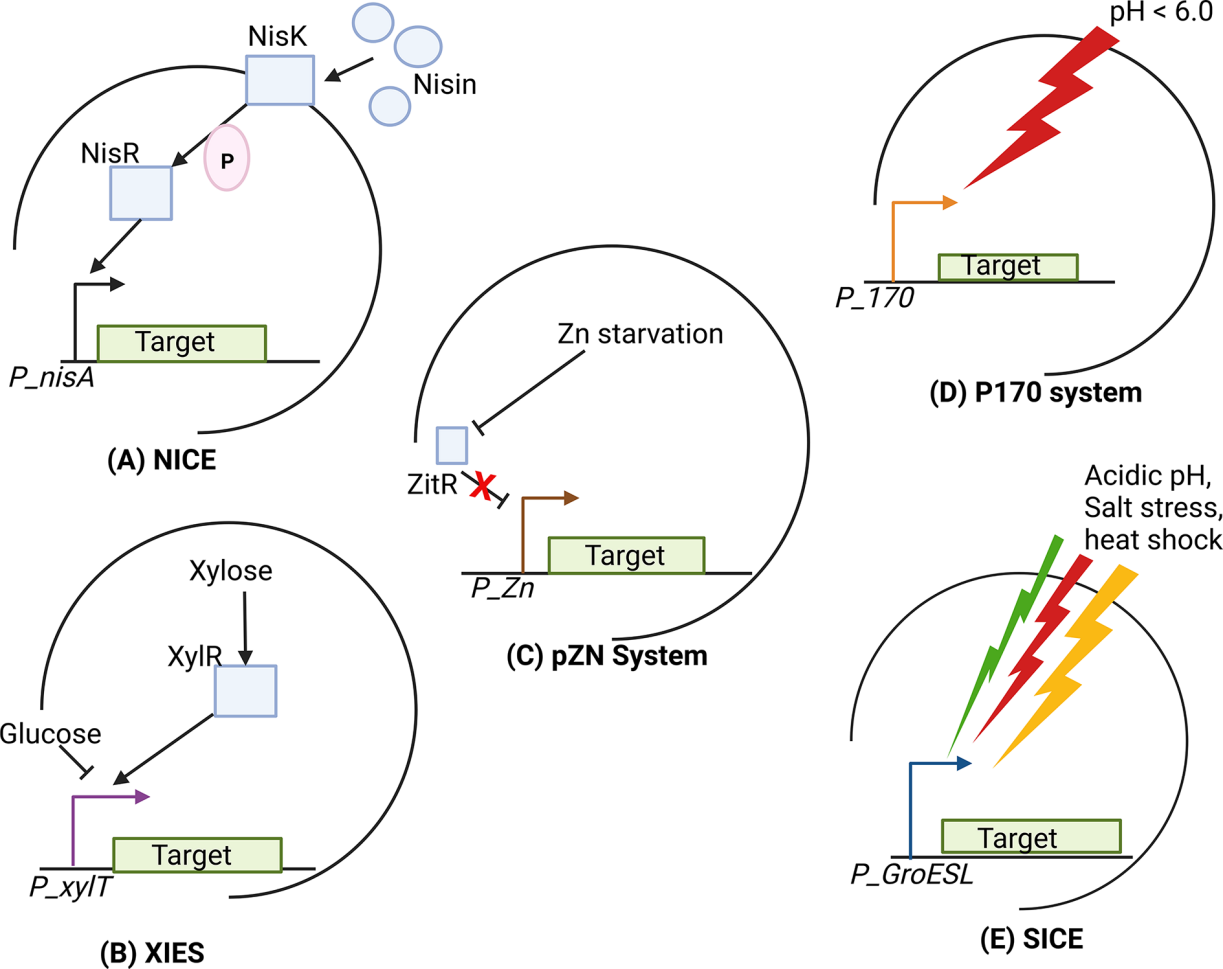
Commonly used promoters in LAB heterologous hosts (**A**) The Nisin-Induced Expression System (NICE), which consists of the sensor protein NisK, the response regulator NisR, and the inducible promoter PnisA. (**B**) The Xylose-Induced Expression System (XIES), which consists of xylose, XylR, glucose, and P_xylT as the inducer, response-regulator, inhibitor, and inducible promoter, respectively. (**C**) The pZn system, which is based on zinc (Zn) starvation, the ZitR response-regulator, and the P_Zn promoter. (**D**) The P170 system, based on the P170 promoter, which is stimulated by an acidic (pH < 6.0) environment. (**E**) The Stress-Induced expression System (SICE). Stresses including acidic pH, salt stress, and heat shock stimulate the GroESL promoter. Stimulation of the promoter drives expression of the target protein. Created with BioRender.com.

The Nisin‐controlled gene expression (NICE) is the most utilised inducible expression system in probiotic engineering [144,145]. This system is used for overexpression of homologous and heterologous proteins, allowing high-level induction of protein expression. The NICE system is typically made up of the histidine kinase (NisK), intracellular response regulator protein (NisR), and the NisA promoter (*PnisA*) (Figure 5) [146]. In the NICE system, nisin is added to the culture medium to stimulate gene expression, and level of induction level correlates positively with the amount of nisin used [141]. The NICE system has been used for heterologous protein production in *Lc. lactis*, *L. helveticus*, and *L. plantarum* (Table 1) [146,147]. The extensive use of the NICE system over the years has demonstrated its versatility and efficiency in heterologous protein expression in LAB.

Another system used for overexpression of heterologous proteins in engineered probiotics is the pSIP system, which is based on quorum sensing approaches [145]. The pSIP system was developed using a series of expression vectors known as the pSIP vectors. These vectors are based on the regulatory genes and promoters of the class II bacteriocins, sakacin A (*sap* gene cluster) or sakacin P (*spp* gene cluster). When using these promoters, gene expression is induced by a peptide pheromone [148]. The pSIP system has been used for induced gene expression in LAB such as *L. casei, Latilactobacillus sakei* (formerly *Lactobacillus sakei*), and *L. plantarum* (Table 1) [149–151].

Llull and Poquet [152] developed a zinc-inducible system for heterologous protein expression in LAB, the P_znZitR system (Figure 5). This system is based on the *Lc. lactis* zit operon, specifically the PZn promoter and zitR repressor (Table 1) [152,153]. The P_znZitR system is strongly inhibited by excess Zn^2+^ in the growth medium and can be induced by zinc depletion [145]. Although this system is highly inducible, it has expression levels about five times lower than those achieved using the NICE system [152]⁠.

Another expression system is the Xylose-Inducible Expression System (XIES), a sugar-dependent system with a 10-fold lower expression level compared to the NICE system (Figure 5). This system is based on the *Lc. lactis* NCDO2118 inducible xylose permease gene promoter (PxylT) [145,154]. The XIES can be activated or repressed by adding either xylose or glucose, respectively [155]. XIES has two versions: a cytoplasmic variant, for production of intracellular proteins, and a secreted variant, for secretion of proteins [156].

One of the most commonly used promoters is the Stress-Induced Controlled Expression (SICE) system (Figure 5). This, unlike other systems, does not require induction or regulatory genes, and is a promising expression system for delivering mucosal therapeutic molecules by LAB (Table 1). The SICE system has been widely used to produce recombinant proteins using *Lc. lactis* [141]. SICE is induced by harsh environmental conditions such as high salt concentration, ultraviolet (UV) irradiation, low pH, or heat shock, and is based on the *Lc. lactis* groESL heat shock protein promoter [155]⁠. Stresses that are encountered by engineered probiotics during GIT transit, such as acid stress, biliary stress, and heat stress, can induce expression in this system and allow the *in situ* production of the heterologous proteins of interest [155].

Another expression system is based on the auto-inducible P170 promoter from *Lc. lactis*. The P170 system is pH inducible and can be activated when lactic acid builds up during the stationary phase, dropping the pH to 6.0 or lower [156] (Figure 5). This system is therefore autoregulated, making it easy to operate, and is a promising promoter controller for heterologous protein production [153]⁠. Other promoters utilised for heterologous expression include the *dnaJ* promoter for heat-shock control [157] and the *sodA* promoter for superoxide dismutase [158].

The use of synthetic promoters for controllable constitutive and inducible gene expression has been reported in *Lc. lactis* and *L. plantarum* [159,160]. Additionally, reporter genes to measure expression of heterologous proteins and for *in vivo* and *in vitro* cell tracking have been developed. Established reporters that have been extensively used in LAB include mCherry, GFP, and beetle red luciferase [159–163].

### Applications of the different expression systems in LAB

The application of expression systems is not only limited to obtaining desired recombinant proteins but is also applied in the development of protein delivery systems [141]. The NICE system was utilised for the expression of anti-HIV lectin griffithsin (GRFT) in *Lacticaseibacillus rhamnosus* (formerly *Lactobacillus rhamnosus*) strains GR-1 and GG, to tackle HIV (Table 1). The GRFT was expressed highly in the cells under the control of the *nisA* promoter [26]. Another study, investigating potential treatments for diabetes and obesity, reported on a NICE-based system for secretion of the recombinant protein, which was constructed in *Lc. lactis* NZ9000. Osteocalcin was secreted into co-cultured medium with mice cells, and subsequently resulted in the stimulation of the mice cells to secrete GLP-1 protein [35]. In efforts to alleviate atherosclerosis, *Lc. lactis* strains expressing recombinant heat-shock protein 65 (HSP65) were constructed and utilised as a mucosal vaccine against atherosclerosis. Expression of the recombinant HSP65 was under the control of the *nisA* promoter. Jing et al. [43] further concluded that engineered LAB are an effective tool for inducing antigen-specific tolerance to tackle autoimmune diseases.

Inducible promoters are commonly employed to provide conditional gene expression, which is useful in therapeutic applications [164]. Inducible systems are useful when overexpression of protein has toxic effects and interferes with the metabolic activity of the host [165]. However, this type of expression system is not suitable for *in situ* production of heterologous proteins in the human body or for applications that require steady-state gene expression. Constitutive promoters are preferred as an alternative in such cases [160]. In a study by Guo et al. [166], a constitutive promoter library was constructed by randomising spacer sequences between the two conserved motifs of the noxE promoter in *Lc. lactis*. Furthermore, activity was monitored for the individual random promoters, and six of the eleven had higher activity than the native promoter. Synthetic promoters to optimise expression have been reported as an alternative method to constitutive expression of heterologous proteins of interest [167]. *Lc. lactis* and *L. plantarum* have been utilised to generate synthetic constitutive promoter libraries, using mutagenesis in promoters [160,165]. Rud et al. [160] developed a synthetic promoter library for *L. plantarum* using a consensus promoter sequence that was derived by aligning rRNA promoters.

Mathipa et al. [5] utilised the expression vector for *Lactobacillus* pLP401T to successfully express internalin AB genes, which are vital for the control of *Listeria monocytogenes*. The pTRKH-IdhGFP backbone expression vector has also been used to construct a human CD4-expressing recombinant vector to engineer *Lactobacillus sp* displaying the HIV-1 receptor protein [168]. Similarly, Van Zyl et al. [100] utilised the pNZ8048 LAB expression vector containing the PnisA inducible promoter to construct integration vectors for use in editing the genomes of *L. plantarum* 423 and *E. mundtii* ST4SA. In another study, Sørvig et al. [151] reported the sakacin P-based vector (pSIP401) and the sakacin A-based vector (pSIP300) as the most suitable for producing GusA in lactobacilli. Later in 2005, the same authors applied pSIP to express high levels of reporter genes, GusA and PepN, in *L. sakei* Lb790 and *L. plantarum* NC8 [169]⁠. Additionally, Kolandaswamy et al. [170] utilised the pSIP401 vector to drive oxalate degrading protein (OxdC) production. Overall, a wide range of expression vectors are available for the expression of heterologous proteins in engineered probiotics.

Several attempts have been made to design probiotics using a diverse combination of tools. The most widely used LAB strain for recombinant protein expression is *Lc. lactis*, which has been engineered to express cytokines, bacterial and viral antigens, membrane proteins, and enzymes. Table 1 shows some of these engineered probiotics and the strategies employed for heterologous protein expression.

### Expression systems for heterologous protein production in engineered probiotics

The heterologous proteins produced by engineered probiotics vary in the type of expression and the location. Engineered probiotics may use an intracellular system (pCYT), a secretion system (pSEC), or a cell wall anchoring system (pCWA) for producing heterologous proteins [145,155]. The location of functional proteins such as antigens, cytokines, and enzymes is particularly important where plasmids are being used for biotherapeutic applications. Engineered probiotics with an intracellular expression system produce and retain heterologous proteins in the cytosol. The protein can only be released by lysing the cell. Other probiotics are engineered with a signal peptide (SP) that allows the secretion production of the heterologous protein. The most widely used signal peptide for the pSEC system is SP_USP_45, which was isolated from an extracellular protein (Usp45) produced by *Lc. lactis* MG1363 (Table 3). Using this system, proteins are synthesised with an N-terminal signal peptide (SP) that ensures their export and translocation [155]. The secretory efficiency of the protein is determined by the nature of the SP. For some applications, such as vaccine development, proteins must be anchored to the cell wall of the recombinant probiotic. The anchoring of heterologous proteins to the bacterial wall is facilitated by the pCWA system. Anchoring systems that are typically used include the lipoprotein anchor [171], the N-terminal transmembrane anchor [172], the non-covalent Lysin Motif domain [30], and the LPxTG peptidoglycan anchor [173].

**Table 3:** Examples of different types of heterologous protein expression systems for engineered probiotics

Expression Type	Examples of engineered strains	Purposes	Reference
**Intracellular (pCYT)**	*Lc. lactis, L. plantarum, L. casei*	Production of antigens and enzymes	[30,43,99,160,174–177]
**Secretion (pSEC)**	*Lc. lactis, L. plantarum, L. helveticus, L. salivarius, L. casei, L. plantarum, L. acidophilus, L. gasseri, L. paracasei*	Secretion of antigens, immunomodulatory proteins, enzymes	[26,33,42,178,179,180]
**Cell wall anchoring (pCWA)**	*Lc. lactis, L. delbrueckii, L. brevis, L. helveticus, L. johnsonii, L. crispatus, and L. salivarius, L. plantarum, Lactobacillus zeae, L. sakei, and L. casei, L. gasseri*	Display of antibodies, antigens, and enzymes	[17,181–183,184–187]

Hugentobler et al. [178] engineered *Lc. lactis* for co-expression of *Leishmania major* antigen (LACK) and IL-12. They demonstrated the use of *Lc. lactis* as an oral live vaccine against *L. major* through heterologous protein expression. This strategy has been employed for the secretion of various antigens and antibodies (Table 3). The use of non-covalent LysM for cell wall anchoring and binding of *Plasmodium* antigen (MSA2) was demonstrated in *Lc. lactis*, *L. sakei*, and *L. casei* by Steen et al. [181]. Similarly, Hu et al. [182] displayed GFP and β-galactosidase on the surfaces of *Lc. lactis*, *Ligilactobacillus salivarius (Lactobacillus salivarius)*, *L. brevis*, *L. crispatus*, *L. johnsonii*, *L. helveticus*, and *L. delbrueckii*, confirming the efficacy of SlpB-mediated surface display. Kuczkowska et al. [183] also developed a vaccine by expressing a lipoprotein-anchored *M. tuberculosis* antigen (AgE6) in *L. brevis*, *L. gasseri, L. plantarum*, and *L. reuteri*. Expression systems continue to be developed for various therapeutic applications. Table 3 summarises the different expression systems for heterologous protein expression.

## Clinical applications of engineered probiotics

The feasibility of using engineered probiotics clinically has been tested using a number of preclinical and clinical trials. For instance, a Phase 1 clinical trial (ClinicalTrials.gov Identifier: NCT04334980) is being conducted to test the immunogenicity of an oral bacTRL-Spike vaccine for the prevention of coronavirus disease 2019 (COVID19). Recombinant *Bifidobacteria longum* expressing bacTRL-Spike, a spike protein, was used to develop the oral vaccine. The vaccine has been shown to induce both cellular and humoral immunity, providing protection against COVID19. In another study, Braat et al. [188] treated patients with Crohn’s disease using recombinant *L. lactis (LL-Thyy12)*, capable of expressing mature human interleukin-10. The efficacy of the recombinant *L. lactis (LL-Thyy12)* was tested in a placebo-uncontrolled trial. They observed a reduction in disease activity and concluded that the use of recombinant bacteria for the mucosal delivery of proteins is feasible in humans. *L. lactis* AG019 was also developed by Precigen ActoBio for the treatment of Type 1 diabetes. Phase 1 and 2 of the clinical trial (ClinicalTrials.gov Identifier: NCT03751007) have been completed. Similarly, Komatsu et al. [189] conducted clinical trials to optimise the mucosal response to recombinant *L. casei* expressing the HPV16 E7 protein. In the second trial, they were able to demonstrate that immunisation with the recombinant vaccine induced the mucosal Th1 immune response. Another clinical study tested the potency of an anti-malarial vaccine produced by recombinant *L. lactis*. The vaccine (GMZ2) is a fusion of two *Plasmodium falciparum* antigens, glutamate-rich protein (GLURP) and merozoite surface protein 3 (MSP3). The trial was carried out in Western, Eastern, and Central Africa with 1849 participants aged between one and five years, and it demonstrated minor efficacy of the GMZ2 antimalarial vaccine in the target population [190]. Most of the clinical trials testing the efficacy of recombinant LAB for various applications have indicated their feasibility as live biotherapeutics. However, there are a number of areas for optimisation, including optimising the expression of heterologous proteins *in situ* and maximising the benefits of the engineered probiotics.

## Safety concerns regarding engineered probiotics

While bioengineered probiotics have a wide range of therapeutic applications, they have a number of drawbacks. One significant drawback is that they are classified as genetically modified organisms (GMOs) and comply with stringent regulations, particularly if they are intended for human consumption. Developing criteria for evaluating environmental safety and monitoring the fate of recombinant probiotics *in vitro* and *in vivo* is challenging. Despite this, probiotic engineering projects must include these criteria in the design phase. A significant biosafety issue that has not yet been resolved is the development of bacterial hosts that cannot live in natural environments.

Biocontainment systems that must be engineered into the genetic construct of probiotics to prevent and/or control the spread of these microorganisms into the environment are guided by GMO regulations. Strategies for biocontainment of engineered probiotics include conditional plasmid replication, auxotrophy, regulation of essential gene expression, and toxin–antitoxin pairs [105]. Gallagher et al. [191] suggested multi-layered safeguards to ensure successful biocontainment of engineered probiotics.

A key challenge in engineering probiotics is ensuring that exogenous DNA is integrated and expressed successfully. Incorporation of exogenous DNA may result in off-site modifications and disruption of essential functions. Off-site modifications may also result in the production of harmful metabolites. Genome modifications in engineered probiotics should therefore take extra precaution to not disrupt the inherent beneficial properties of the organism or result in the production of toxic metabolites in organisms that have traditionally been Generally Regarded as Safe (GRAS).

For probiotic engineering to fully realise its potential, the safety of bioengineered probiotics must be guaranteed.

## Conclusions and perspectives

There have been great strides in engineering probiotics for various therapeutic applications. Several tools are available for engineering probiotics and many more continue to be developed. However, the effectiveness of genetic engineering tools has been shown to be strain or species specific. Standardised engineering tool sets must be developed to ensure broad applicability of these tools to readily engineer any probiotic.

Key considerations in engineering probiotics include the inherent qualities of the chassis organism, suitability of the chassis for heterologous protein expression, accessibility of the microorganism to genome editing tools, and safety. An approach to engineering probiotic LAB that has not been widely explored is the minimal genome approach. This approach has a lot of potential in improving the efficiency of heterologous protein production by engineered LAB. Efforts should be made to use computational biology tools and available empirical data to develop whole-cell models for rational design of probiotic LAB. This would likely provide better accuracy in determining essential and non-essential genes in the genomes of probiotic engineering targets. Additionally, whole-cell models would allow accurate predictions of the impact of genome minimisation under simulated environmental conditions. Using a computational biology approach together with the minimal genome approach is likely to improve the efficiency of probiotic engineering.

In future, we anticipate that probiotics will be engineered to develop novel therapeutic approaches. A promising approach is to develop probiotic chassis for the delivery of vaccines against infectious diseases and cancers. Most of the studies in this review evaluated heterologous protein production by engineered probiotics using *in vitro* assays or animal models. To advance probiotic engineering efforts, more *in vivo* and clinical trials should be done to validate the effectiveness of heterologous protein expression and associated therapeutic applications in human health. In order to address these unmet need, we at CSIR are currently working on Lactochassis where we develop synthetic LAB using synthetic biology tools.

## Abbreviations

GIT: gastrointestinal tract
GLURP: glutamate-rich protein
GMO: genetically modified organism
GRAS: Generally Regarded as Safe
HSP65: heat-shock protein 65
LAB: lactic acid bacteria
MSP3: merozoite surface protein 3
PAM: protospacer adjacent motif
SICE: Stress-Induced Controlled Expression
